# Cox Models: Lepeule et al. Respond

**Published:** 2006-12

**Authors:** Johanna Lepeule, Virginie Rondeau, Jean-François Dartigues, Laurent Filleul

**Affiliations:** INSERM, EMI 0338 (Biostatistique), Université Victor Segalen, Bordeaux, France, E-mail: lepeule@vet-nantes.fr; INSERM, U 593, Université Victor Segalen, Bordeaux, France; Institut de Veille Sanitaire - CIRE Aquitaine, Bordeaux, France

We read with interest the letter by Lumley et al. regarding our article (Lepeule et al. 2006), and we appreciate their comments and interesting suggestions.

Our results (Lepeule et al. 2006) showed that the Cox model (Cox 1972) approach gave more precise results for cohort data than the case-crossover design (Maclure 1991). As stated by Lumley et al., the Cox model is more efficient than the semisymmetric bidirectional case-crossover design because more referent time points are used. In fact, because the case-crossover design is a within-people approach, people who do not have the event are not included in the analysis, whereas they are in the Cox model and so contribute to the information for estimating the exposure effect.

Lumley et al. specify that the estimating equations for the Cox regression are the same as those used in the conditional logistic regression for the case-crossover design and that we applied them to the same data. Despite that, we cannot say that the Cox model is a case-crossover design with an unusual choice of referent strategy. As we stated in our article (Lepeule et al. 2006), the results of both approaches are very similar, and when a cohort is available, the Cox model should be applied because survival analysis uses all available information and increases the power of the study. The case-crossover analysis is a within-person approach; the referent time points are chosen by the operator, and the design is the same for all the subjects. The Cox model is a between-people approach; the referent time points cannot be chosen because they depend on the number of live subjects who will be included in the risk set, which varies at each time of death. Moreover, with age used as the basic time scale, the dispersion of the referent time points included in the risk set around the time of death varies at each age of death. Otherwise, the number of referent time points is almost always higher in the Cox model than in the case-crossover design (i.e., two referent time points in the bidirectional design).

We do not agree with the statement of Lumley et al. that

They choseβ; thus, the exposure for an individual who died at a given age is equal to the average exposure for at-risk individuals at exactly that age.

In fact, we assess β as the exposure for a person who died at a given age compared with the exposures for at-risk people at exactly that age: β is the mean effect for an increase in air pollution concentration on the mortality, whatever the age. Thus, in both cases, when the exposure is either a chronic measurement or an ecologic time-series data set, the Cox model captures all of the information available, whereas the case-crossover design cannot be used with chronic exposure measurements. Therefore, the Cox model should prove particularly useful in the future to simultaneously analyze both the chronic (long-term) and the short-term effects of air pollution concentrations.

Concerning the first possible bias noted by Lumley et al., the adjustment of the results for the seasonality effect and for time trends in air pollution concentration is more of an advantage than a disadvantage. These pieces of information are very easy to take into account with truncated power basis splines (Heuer 1997) without data collection. Moreover, this process allows for the assessment of the magnitude of these effects, which is not possible with the case-crossover design. The second bias noted by Lumley et al. on the extreme age of death is a very minor bias that was not present in our study. This bias appears only if there is no risk set for the first or the last subject who has the event.

Furthermore, numerous results from time-series studies have shown an association between mortality and particulate air pollution, and the results observed were similar (Filleul et al. 2001; Goldberg et al. 2001; Samet et al. 2000). Despite that, causality was discussed (Filleul et al. 2003) and statistical methods have sometimes been criticized. For example, generalized additive models using nonparametric smoothing, which could lead to biased estimates and to underestimation of the true variance (Dominici et al. 2002, Ramsay et al. 2003). Thus, using the Cox model could be an alternative approach if data are available.

Our study (Lepeule et al. 2006) is the first in which a Cox model has been used to study the short-term effect of air pollution. We found that the Cox method and case-crossover design gave the same results as times series. This information supports the hypothesis of a causal relationship between mortality and air pollution.
